# Metagenomic analysis revealed the distribution of antibiotic resistance genes of Awang sheep (*Ovis aries*) gut microbiota

**DOI:** 10.3389/fmicb.2025.1740198

**Published:** 2026-01-14

**Authors:** Siyue Zhao, Xinping Wang, Heran Zhu, Ge Guo, Ghulam Raza Mustafa, Ahsan Mustafa, Yu Chen, Xiangle Li, Ying Wang, Bi Zhao

**Affiliations:** 1 College of Water Conservancy and Hydropower Engineering, Sichuan Agricultural University, Yaan, Sichuan, China; 2 Xizang Changdu Animal Husbandry Station, Changdu, Xizang, China; 3 Yunnan Tropical and Subtropical Animal Virus Disease Laboratory, Yunnan Academy of Animal Husbandry and Veterinary Sciences, Kunming, Yunnan, China; 4 College of Animal Science, Shandong Agricultural University, Taian, Shandong, China; 5 Tea Research Institute, Yunnan Academy of Agricultural Sciences, Yunnan Key Laboratory of Tea Science, Kunming, Yunnan, China

**Keywords:** antibiotics, Awang sheep, gut microbiota, resistance genes, Tibet

## Abstract

Antimicrobial resistance (AMR) in livestock is a major contributor to the global AMR crisis, yet little is known about its dynamics in high-altitude pastoral systems. We performed deep metagenomic sequencing of 100 fecal samples from Tibetan Awang sheep reared under grazing (aw_fm) and captive (aw_qs) conditions. Microbiome profiling revealed striking community shifts: grazing sheep were enriched in Bacteroidetes and Firmicutes, whereas captive sheep showed expansion of Proteobacteria, particularly *Acinetobacter*, suggesting dysbiosis. The resistome comprised 302 unique ARGs, dominated by *rpoB2* (43.3%), *Bifidobacterium*_*adolescentis*_*rpoB* (11.2%), and *ugd* (10.2%). Grazing sheep carried ARGs mainly against rifamycins and peptide antibiotics, reflecting natural selective pressures, while captive sheep exhibited significantly broader resistance, including tetracyclines, macrolides, and fluoroquinolones (*p* < 0.05). Enrichment of efflux pump genes (*MexK*, *adeJ*) in captive sheep highlighted a shift toward multidrug resistance. These findings demonstrate that rearing practices profoundly restructure the gut resistome, underscoring the need for targeted antibiotic stewardship in high-altitude livestock systems.

## Highlights

- Microbiome divergence by rearing system: Grazing sheep harbor fiber-degrading *Bacteroidetes*, whereas confinement favors opportunistic *Acinetobacter* and enrichment of clinical ARGs (*MexK/adeJ*).
- Natural vs. anthropogenic ARGs: *rpoB2* was dominated by plant-associated resistance genes, such as *rpoB2*, while confinement herds harbor high-risk, plasmid-borne resistance determinants against tetracycline/macrolide genes.
- One health significance: Intensive livestock management accelerates the emergence and dissemination of AMR, underscoring the urgency of targeted interventions, such as feed reformulation and antibiotic stewardship, to limit resistome transmission across environments.

## 1 Introduction

Antimicrobial resistance (AMR) has emerged as one of the most pressing global health threats, undermining the efficacy of antibacterial therapies as pathogens acquire and disseminate resistance determinants (Kariuki, 2024). Livestock production systems contribute substantially to this crisis, accounting for approximately 70% of global antimicrobial consumption (Van Boeckel et al., 2017). The animal gut microbiome represents a particularly important reservoir of antibiotic resistance genes (ARGs), where dense and diverse microbial communities promote horizontal gene transfer (HGT) via mobile genetic elements (MGEs), enabling the spread of resistance from commensals to pathogens (Hu et al., 2013). Mounting evidence indicates that livestock-associated ARGs can cross ecological boundaries, reaching humans through direct contact, food production chains, and environmental pathways (Larsson and Flach, 2022).

The sheep gut harbors an exceptionally dense and diverse microbiota, creating a hotspot for ARG acquisition and transfer (Kamke et al., 2016). Notably, comparative analyses reveal striking geographical variation in sheep resistomes: European flocks are dominated by tetracycline resistance genes (*tetW*, *tetO*), while Chinese breeds exhibit elevated *β*-lactamase abundances (*bla*_TEM_, *bla*_CTX-M_), reflecting local antibiotic usage practices (Zeng et al., 2019). Such divergence highlights the importance of breed- and region-specific resistome profiling, particularly for indigenous varieties such as Tibetan Awang sheep that remain underexplored despite their ecological and agricultural significance.

The Tibetan Awang sheep (*Ovis aries*), an indigenous breed endemic to the Qinghai-Tibet Plateau, offer a unique model for resistome ecology. Adapted to hypoxic, nutrient-poor environments, Awang sheep possess an enlarged rumen (15–20% greater volume than lowland breeds), which enhances fermentation capacity and supports distinct microbial assemblages (Fan et al., 2021). Traditional grazing practices further expose these sheep to diverse environmental microbial reservoirs, including soil and waterborne ARG carriers (Sabino et al., 2019). Combined with prolonged digestion retention and high microbial density, these factors may impose novel selective pressures on the gut resistome. Moreover, high-altitude ruminants are hypothesized to harbor efflux pump-associated ARGs, potentially linked to detoxification of plant secondary metabolites, a phenomenon previously observed in plateau-adapted livestock (Zeng et al., 2019).

Despite these unique ecological and physiological features, the resistome of Awang sheep remains uncharacterized. Here, we present, to our knowledge, the first metagenomic survey of ARG diversity and abundance in this high-altitude breed. By comparing grazing and captive populations, we provide critical insights into how rearing conditions and environmental exposures shape the gut resistome. This study establishes a baseline framework for AMR surveillance in indigenous Chinese sheep and informs targeted antibiotic stewardship strategies tailored to high-altitude pastoral systems.

## 2 Methods

### 2.1 Sample collection

**Figure 1:**
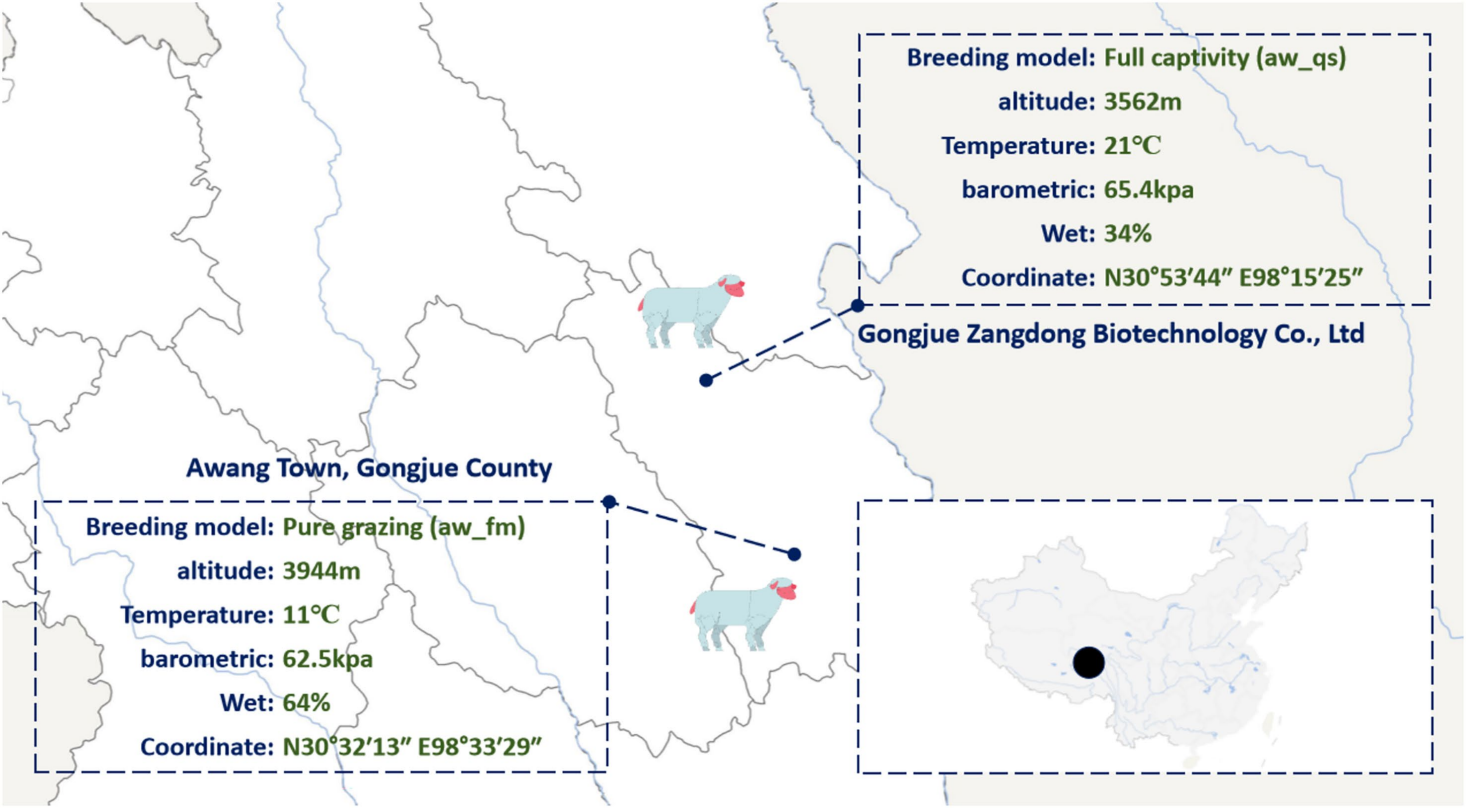
Sampling map.

Fecal samplings were collected on 5 July 2023, with approval from the Xizang Changdu Animal Husbandry Station (China) and in compliance with institutional ethical guidelines. Sampling was conducted at two bases: Gongjue County, Tibet (30°54′N, 98°52′E) and Gongjue Zangdong Biotechnology Co., Ltd. (30°89′N, 98°26′E) (Figure 1). Fresh fecal material was obtained immediately after defecation from 100 healthy Awang sheep using sterile 50 mL tubes, snap-frozen in liquid nitrogen, and transported on dry ice to the laboratory, where samples were stored at −80 °C until processing. The samples were categorized into two breeding models: pure grazing (aw_fm, *n* = 50) and full captivity (aw_qs, *n* = 50). All individuals were 2-year-old males, selected to minimize age- and sex-related confounding. Pure grazing sheep (25–30 kg) were maintained exclusively on natural meadow forage, whereas full captivity sheep (27–33 kg) were fed concentrate diets. Veterinary inspection confirmed that all animals were clinically healthy at the time of sampling. Metadata, including body weight and feeding regime, are provided in Supplementary Table S1.

### 2.2 DNA isolation and library construction

The total genomic DNA from the samples was extracted using the QIAamp® PowerFecal® Pro DNA Kit (Qiagen, Inc., Germany) according to the manufacturer’s instructions. DNA concentration and integrity were assessed by a NanoDrop2000 spectrophotometer (Thermo Fisher Scientific, Waltham, MA, USA) and agarose gel electrophoresis, respectively. DNA was fragmented by S220 Focused-ultrasonicators (Covaris, USA) and purified with Agencourt AMPure XP beads (Beckman Coulter Co., USA). Then, libraries were constructed using the TruSeq Nano DNA LT Sample Preparation Kit (Illumina, San Diego, CA, USA) according to the manufacturer’s instructions. Metagenome sequencing and analysis were conducted by OE Biotech Co., Ltd. (Shanghai, China).

### 2.3 Bioinformatic analysis

The libraries were sequenced on an Illumina NovaSeq6000 platform, and 150 bp paired-end reads were generated. Sequences in the FastQ file were trimmed and filtered using fastp (v 0.20.1) (Chen et al., 2018). Host pollution control was needed. The post-filtered paired-end reads were aligned against the host genome using bbmap (v 38.93–0), and the aligned reads were discarded. Metagenome assembly was performed using MEGAHIT (v 1.2.9) (Kamke et al., 2016; Li et al., 2015) after getting valid reads. Using gaps inside the scaffold as a breakpoint to interrupt the scaffold into new contigs (Scaftigs), and these new Scaftigs with length> 500 bp were retained. ORF prediction of assembled scaffolds using prodigal (v 2.6.3) (Buchfink et al., 2015) was performed and translated into amino acid sequences. The non-redundant gene sets were built for all predicted genes using MMSeqs2 (v 13.45111). The clustering parameters were 95% identity and 90% coverage. The longest gene was selected as the representative sequence of each gene set. Clean reads of each sample were aligned against the non-redundant gene set (95% identity) using salmon (v 1.8.0), and the abundant information of the gene in the corresponding sample was counted. The taxonomy of the species was obtained as a result of the corresponding taxonomy database of the NR Library. To construct the abundance profile on the corresponding taxonomy level, abundance statistics were performed at each level of domain, kingdom, phylum, class, order, family, genus, and species. The gene set representative sequence (amino acid sequence) was annotated with NR, KEGG (Kanehisa et al., 2014; Powell et al., 2014), eggNOG (Hyatt et al., 2010), SWISSPROT, and the GO database with an e-value of 1e-5 using DIAMOND (v 0.9.10.111) (Cantarel et al., 2009). The gene sets were compared with the CAZy database (Kanehisa et al., 2006) using the corresponding tool hmmscan (v 3.1) to obtain information about the carbohydrate-active enzyme corresponding to the gene, and then the carbohydrate activity was calculated using the sum of the gene abundances corresponding to the carbohydrate-active enzyme abundance. The PCA analysis and plotting of the taxonomy abundance spectrum or functional abundance spectrum were carried out using R software (v 4.1.2), and the results of the equidistant matrix of PCoA and NMDS were calculated and analyzed. Then the R package was used to analyze the significant differences between different groups using the ANOVA statistical test. The linear discriminant analysis effect size (LEfSe) method was used to compare the taxonomy abundance spectrum and functional abundance spectrum.

### 2.4 Statistical analysis and data visualization

Most of the subsequent statistical analysis was performed using Microsoft Excel (Microsoft Inc.), Python,^1^ and R (package: ggplot2 and vegan).^2^ Indices of gut bacterial richness (Chao1 index) and diversity (Shannon index) were calculated using software R^3^ for alpha diversity analysis. The T-test method was used for statistical differences among groups. For beta diversity, the principal coordinate analysis (PCoA) was performed based on the ASV-based weighted Unifrac and Bray–Curtis distance matrices using R software^4^ with the GUniFrac, ape, and ggplot2 packages (Jiang et al., 2013). A one-way analysis of similarity (ANOSIM) (Li et al., 2017) was conducted to assess the differences in beta diversity among all the breeding models of Awang sheep. The specific species that had significant differences at each level were identified and visualized through LDA effect size (LEfSe) analysis performed online.^5^

## 3 Results

### 3.1 Overview of the sequencing dataset

A total of 100 gut metagenomes (50 grazing, *aw_fm*, 50 captives, *aw_qs*) were subjected to the Illumina NovaSeq platform (paired-end, 2 × 150 bp), generating ~1.45 terabases (Tb) of raw data. Following stringent quality filtering, approximately 10 billion high-quality reads were retained, corresponding to an average of 14.6 ± 3.2 Gb per sample. *De novo* assembly with MEGAHIT (v1.2.9, k-mer range 21–121) produced 1,969,590 contigs ≥ 500 bp in length on average. Comprehensive assembly and sequencing statistics for each sample are summarized in Supplementary Table S2.

### 3.2 Bacterial diversity and composition

**Figure 2:**
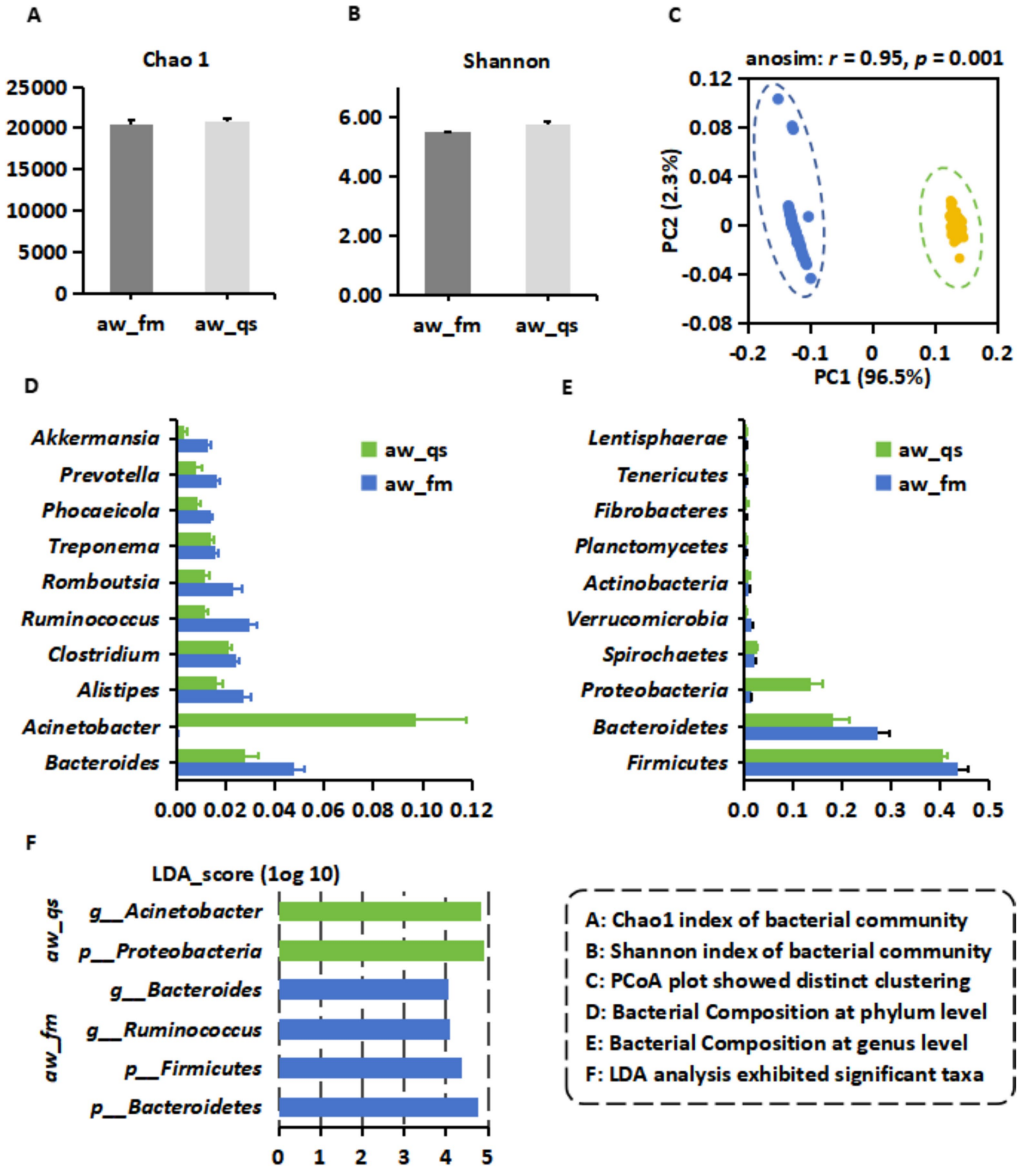
Comparative analysis of gut microbiota composition of grazing and captive Awang sheep. It includes six key comments: **(A)** Chao1 index of the bacterial community. **(B)** Shannon index of bacterial community. **(C)** PCoA plot shows distinct clustering. **(D)** Bacterial composition at the phylum level. **(E)** Bacterial composition at the genus level. **(F)** LDA analysis exhibited significant taxa.

We compared the gut microbiota of grazing (*aw_fm*) and captive (*aw_qs*) Awang sheep using alpha and beta diversity analyses. Alpha diversity, assessed by the Chao1 (richness) and Shannon (evenness) indices, showed no significant differences between the two groups (Chao1: 20,490 ± 526 vs. 20,781 ± 358; Shannon: 5.47 ± 0.03 vs. 5.76 ± 0.09; *p* > 0.05) (Figures 2A–B). In contrast, beta diversity revealed clear group-specific separation: principal coordinate analysis (PCoA) demonstrated a distinct clustering of samples by rearing condition (Figure 2C), a pattern further supported by ANOSIM (r = 0.95, *p* < 0.001), indicating significant compositional differences between groups.

Taxonomic profiling identified 39 ± 0 phyla across both groups, with 2,434 ± 41 genera detected in the grazing sheep and 2,399 ± 25 genera in the captivity sheep. At the phylum level, Firmicutes is the predominant phylum in both cohorts (43.7% ± 2% in the grazing, 40.5% ± 1% in captivity), followed by Bacteroidetes (27.2% ± 2.6% vs. 18.1% ± 3.4%, respectively). Notably, Proteobacteria were markedly enriched in captive sheep (13.4% ± 2.6%), emerging as the third most abundant phylum (Figure 2D). At the genus level, grazing sheep were dominated by *Bacteroides* (4.8% ± 0.4%), *Ruminococcus* (2.9% ± 0.3%), *Alistipes* (2.7% ± 0.3%), and *Clostridium* (2.4% ± 0.1%). In contrast, captive sheep exhibited a striking dominance of *Acinetobacter* (9.7% ± 2%), followed by *Bacteroides* (2.8% ± 0.5%), *Clostridium* (2.1% ± 0.1%), *Alistipes* (1.6% ± 0.3%), and *Ruminococcus* (1.2% ± 0.1%) (Figure 2E).

LEfSe analysis (LDA score > 4, *p* < 0.001) confirmed these compositional differences. Grazing sheep were enriched in Bacteroidetes and Firmicutes at the phylum level and in *Ruminococcus* and *Bacteroides* at the genus level, whereas captive sheep exhibited significant enrichment in Proteobacteria and *Acinetobacter* (Figure 2F). Collectively, these findings highlight a diet- and environment-driven divergence in the gut microbial structure, with confinement strongly associated with Proteobacteria expansion and opportunistic pathogen dominance.

### 3.3 Distribution of resistance mechanisms, antibiotics, and ARGs in the Awang sheep gut microbiota

#### 3.3.1 Overall ARG distribution profile

**Figure 3:**
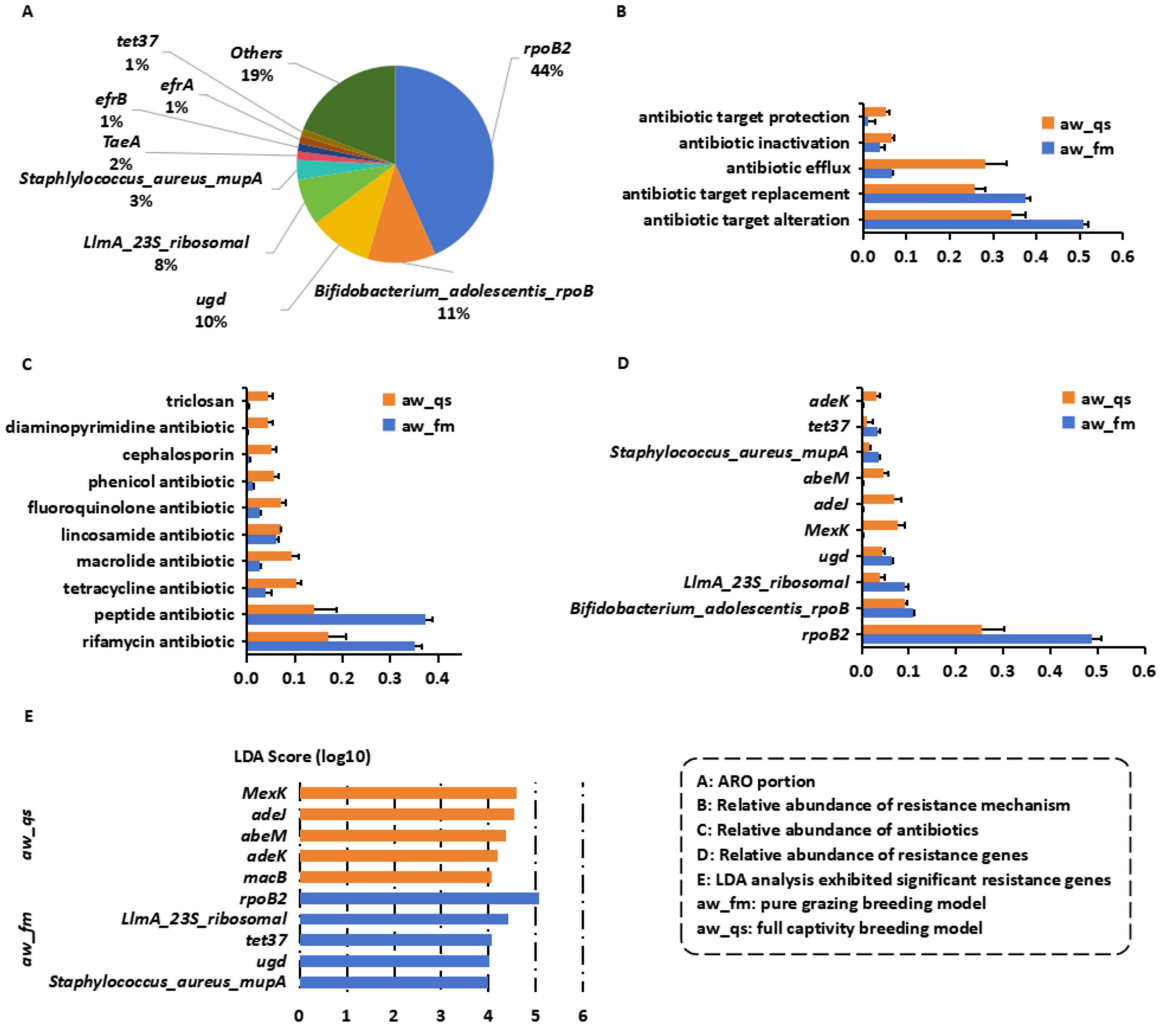
Distribution and characterization of antibiotic resistance genes (ARGs) in the gut microbiota of grazing and captive Awang sheep. It includes five key comments: **(A)** Overall ARG distribution profile in Awang sheep gut microbiota. **(B)** Resistance mechanisms in different rearing systems. **(C)** Antibiotic resistance potential of ARGs. **(D)** Dominant ARGs in grazing vs. captive sheep. **(E)** Differential ARG enrichment between grazing and captive groups.

Metagenomic analysis identified a total of 302 unique antibiotic resistance genes (ARGs) in the gut microbiota of Awang sheep. The most abundant ARG was *rpoB2* (5,341, 43.3%), followed by *Bifidobacterium_adolescentis_rpoB* (1,385, 11.2%), *ugd* (1,266, 10.2%), *LlmA_23S_ribosomal* (939, 7.6%), and *Staphlylococcus_aureus_mupA* (398, 3.2%). Additional but less abundant genes included *TaeA*, *efrB*, *efrA*, and *tet37* (Figure 3A).

#### 3.3.2 Resistance mechanisms in different rearing systems

ARGs were classified into five major resistance mechanisms (Figure 3B). In grazing sheep (aw_fm), the resistome was dominated by antibiotic target alteration (50.8% ± 1.2%) and target replacement (37.5% ± 1.1%), with minor contributions from efflux (6.5% ± 0.5%), inactivation (3.8% ± 1.1%), and target protection (1.2% ± 1.4%). In contrast, captive sheep (aw_qs) exhibited a striking shift: while target alteration (34.1% ± 3.2%) and target replacement (25.8% ± 2.4%) remained prominent, efflux pump–mediated resistance increased significantly (28.2% ± 4.9%), indicating a transition toward multidrug resistance mechanisms.

#### 3.3.3 Antibiotic resistance potential of ARGs

ARGs in grazing sheep primarily conferred resistance to rifamycins (35% ± 1.5%) and peptide antibiotics (37.3% ± 1.4%), with lower levels against tetracyclines (4% ± 1.2%), macrolides (2.6% ± 0.2%), and fluoroquinolones (2.6% ± 0.3%) (Figure 3C). In contrast, captive sheep harbored a broad resistance spectrum, with significantly elevated proportions of tetracycline (10.5% ± 1%), macrolide (9.5% ± 1.5%), fluoroquinolone (7.1% ± 1.1%), and cephalosporin (5.2% ± 1.1%) resistance genes (*p* < 0.05).

#### 3.3.4 Dominant ARGs in grazing vs. captive sheep

In grazing sheep, *rpoB2* overwhelmingly dominated the resistome*2* (48.8% ± 2.1%), followed by *Bifidobacterium_adolescentis_rpoB* (10.8% ± 0.4%), *LlmA_23S_ribosomal* (9.2% ± 0.6%), *ugd* (6.4% ± 0.2%), *Staphylococcus_aureus_mupA* (3.5% ± 0.3%), and *tet37* (3.4% ± 0.4%) (Figure 3D). By contrast, the relative abundance of *rpoB2* decreased markedly in captive sheep (25.5% ± 4.6%), accompanied by significant enrichment of efflux pump-associated genes, including *MexK* (7.7% ± 1.6%), *adeJ* (6.9% ± 1.4%), and *abeM* (4.6% ± 1.1%). These differences indicate that while natural grazing systems favor ARGs linked to environmental antibiotic exposure, confinement selects for multidrug efflux systems commonly associated with clinical resistance.

#### 3.3.5 Differential ARG enrichment between groups

LEfSe analysis (LDA score > 4, *p* < 0.001), further confirmed distinct ARG signatures (Figure 3E). Grazing sheep were enriched with *rpoB2*, *LlmA_23S_ribosomal*, *tet37*, *ugd*, and *Staphylococcus_aureus_mupA*. In contrast, captive sheep were enriched in efflux-related genes (*MexK*, *adeJ*, *abeM*, *adeK*, and *macB*), highlighting confinement-driven selection for multidrug resistance determinants. These results demonstrate that while grazing systems are dominated by natural resistance genes associated with environmental exposure, confinement fosters a resistome enriched in broad-spectrum and efflux-mediated ARGs, reflecting anthropogenic selective pressures with direct implications for antimicrobial resistance dissemination.

### 3.4 Host–microbiome origin of dominant ARGs

Metagenomic host assignment revealed that the dominant ARGs originated from diverse bacterial taxa with distinct resistance spectra (Table 1). The highly abundant *rpoB2* gene was primarily associated with *Nocardia farcinica IFM 10152*, conferring resistance through both antibiotic target alteration and replacement. Similarly, *Bifidobacterium_adolescentis_rpoB* derived from *Bifidobacterium_adolescentis* also mediated rifamycin resistance via analogous mechanisms. Other functionally important ARGs included *LlmA_23S_ribosomal* from *Paenibacillus* sp. *LC231,* which conferred resistance to lincosamide through target alteration, and *ugd* from *Escherichia coli str. K-12 MG1655,* which mediates resistance to peptide antibiotics via target modification.

**Table 1:** Dominant antibiotic resistance gene catalog.

Resistant genes	Antibiotics	Resistance mechanism	Source of taxa
*rpoB2*	Rifamycin	Antibiotic target alteration Antibiotic target replacement	*Nocardia farcinica IFM 10152*
*Bifidobacterium_adolescentis_rpoB*	Rifamycin	Antibiotic target alteration Antibiotic target replacement	*Bifidobacterium_adolescentis*
*LlmA_23S_ribosomal*	Lincosamide	Antibiotic target alteration	*Paenibacillus* sp. *LC231*
*Ugd*	Peptide	Antibiotic target alteration	*Escherichia coli str. K-12 substr. MG1655*
*MexK*	Macrolide Tetracycline Triclosan	Antibiotic efflux	*Pseudomonas aeruginosa PAO1*
*adeJ*	Carbapenem Cephalosporin Diaminopyrimidine Fluoroquinolone Lincosamide Macrolide Penem Phenicol Rifamycin Tetracycline	Antibiotic efflux	*Acinetobacter baumannii*
*AbeM*	Acridine Fluoroquinolone Triclosan	Antibiotic efflux	*Acinetobacter baumannii*
*Staphylococcus_aureus_mupA*	Mupirocin	Antibiotic target alteration	*Staphylococcus aureus*
*tet37*	Tetracycline	Antibiotic inactivation	None

In contrast, confinement-enriched ARGs were dominated by multidrug efflux systems. Specifically, *MexK* from *Pseudomonas aeruginosa PAO1* conferred resistance to macrolide, tetracycline, and triclosan, while *adeJ* and *abeM* from *Acinetobacter baumannii* mediated broad resistance phenotypes. The *adeJ* exhibited one of the widest resistance ranges, spanning carbapenem, cephalosporin, macrolide, penem, phenicols, rifamycin, and tetracycline. While *abeM* was associated with resistance to acridines, fluoroquinolones, and triclosan. Notably, *Staphylococcus_aureus_mupA* derived from *Staphylococcus aureus,* conferred mupirocin resistance via target alteration, and *tet37,* although host assignment was unresolved, mediated tetracycline inactivation.

## 4 Discussion

The metagenomic sequencing of 100 gut samples generated an exceptionally deep dataset (~1.45 Tb raw data, ~10 billion high-quality reads), providing robust resolution of microbial community dynamics and antibiotic resistance genes (ARGs) distribution in Awang sheep. The high sequencing depth (>14.6 Gb per sample) ensured capture of rare taxa and low-abundance ARGs, which are increasingly recognized as functionally pivotal in gut ecosystems (Ye et al., 2019). Such coverage is particularly important given the complexity of the gut microbiota; minor taxa may disproportionately contribute to digestion, metabolic adaptation, and resistome evolution.

Although alpha diversity (Chao1, Shannon) showed no significant differences between grazing (aw_fm) and captive (aw_qs) sheep, clear beta diversity separation (ANOSIM: *r* = 0.95, *p* < 0.001) revealed strong compositional shifts driven by feeding regimen. Firmicutes predominated across both groups, consistent with their established role in fiber degradation and short-chain fatty acid (SCFA) production (Myer et al., 2015). Grazing sheep exhibited higher Bacteroidetes abundance (27.2% vs. 18.1%), likely reflecting specialization in complex polysaccharide metabolism inherent to forage-based diets (Henderson et al., 2015). Conversely, captivity enriched Proteobacteria (13.4%), dominated by Acinetobacter (9.7%), a gene associated with gut dysbiosis and inflammation in livestock (Lima et al., 2024). Elevated Proteobacteria have been linked to impaired suboptimal fiber digestibility, stress, or antibiotic exposure (Shin et al., 2015), suggesting that confinement stressors or dietary additives (e.g., growth promoters) may favor opportunistic taxa, mirroring patterns observed in intensively reared poultry (Campos et al., 2025). LEfSe analysis further reinforced functional divergence, with grazing sheep enriched in *Ruminococcus* and *Bacteroides*, key cellulolytic and hemicellulolytic taxa (Biddle et al., 2013). While *Acinetobacter* emerged as the dominant taxon in captivity. This is particularly concerning, as *Acinetobacter* is a known reservoir for multidrug-resistant plasmids (Kornelsen and Kumar, 2021). These findings highlight how feeding models shape gut ecology, with potential implications for host health, digestion efficiency, and resistome dynamics.

Resistance mechanisms also diverged between feeding regimens. Target alteration was the dominant mechanism in both groups (50.8% in aw_fm; 34.1% in aw_qs), consistent with its role as a stable evolutionary strategy under low-level selective pressures (Wright, 2011). However, the efflux pump was markedly higher in captive (28.2% vs. 6.5%), suggesting dietary or environmental exposures that favored multidrug resistance strategies. Plant-derived secondary metabolites, such as tannins and flavonoids, known inducers of efflux pump (Blanco et al., 2016), may contribute to this pattern, while horizontal gene transfer (HGT) from environmental bacteria could further amplify efflux-associated resistance (Von Wintersdorff et al., 2016). The inverse relationship between target alteration (a reduction of 16.7% in captivity) and efflux activity (an increase of 21.7%) suggests a potential ecological trade-off; while target alteration offers stable, mutation-driven resistance, efflux provides broader coverage but a higher energetic cost (Andersson and Hughes, 2010). This dynamic implies differential selective pressures—grazing may favor persistent genomic adaptations, whereas captivity imposes intermittent multi-drug pressures favoring efflux.

The predominance of *rpoB2* (43.3%), encoding an RNA polymerase *β*-subunit mutation conferring rifampicin resistance, underscores strong selective pressure. Although rifampicin is rarely used in livestock, cross-resistance to plant-derived RNA polymerase inhibitors such as salicylates or phenolics may explain its high abundance in grazing sheep (Goldstein, 2014). The presence of *Bifidobacterium_adolescentis_rpoB* (11.2%) further supports this hypothesis, as *Bifidobacterium* spp. metabolize dietary polyphenols, potentially co-selecting for *rpoB* mutations (Grimm et al., 2015). However, it is important to note that *Bifidobacterium* spp. were not directly detected in our samples. This discrepancy suggests that the assignment may not be fully accurate and requires further validation. Future studies should aim to clarify this assignment through additional experimental approaches.

The detection of *ugd* (10.2%), linked to polymyxin resistance via lipid A modification, likely reflects adaptation to endogenous antimicrobial peptides (e.g., defensins), rather than clinical polymyxin exposure (Olaitan et al., 2014). Efflux-associated genes such as *MexK* (*Pseudomonas aeruginosa*) and *adeJ*/*abeM* (*Acinetobacter baumannii*) were enriched in captivity, consistent with anthropogenic antibiotic exposure and subtherapeutic use in intensive farming systems (Zhao et al., 2021). The detection of *mupA* in 3.2% of grazing sheep is particularly intriguing, considering that mupirocin is primarily used in human medicine (Poovelikunnel et al., 2015). This observation leads us to hypothesize several potential sources for this resistance gene in sheep. One hypothesis is anthropogenic contamination, where environmental exposure to human waste or runoff containing mupirocin residues could introduce the gene into the grazing ecosystem. For instance, studies have shown that human activities can lead to the dissemination of antibiotics in agricultural settings (Kummerer, 2009). Another potential hypothesis is the undocumented off-label use of mupirocin in veterinary medicine, which may occur due to a lack of regulation or oversight, although there is limited evidence to support this practice currently (Fromberg et al., 2017). These hypotheses underscore the need for further investigation, including environmental sampling and interviews with local farmers, to elucidate the pathways through which *mupA* is introduced and maintained in these populations. Future studies should aim to clarify these mechanisms to better understand the implications for One Health and antimicrobial resistance management.

Resistance profiles revealed contrasting selection pressures: grazing sheep were dominated by rifamycin (35%) and peptide (37.3%) resistance, likely reflecting adaptation to soil-derived antibiotics and host antimicrobial peptides (AMPs) (Donia and Fischbach, 2015). In contrast, captive sheep exhibited broader resistance spectra, with elevated tetracyclines (10.5%), macrolides (9.5%), and fluoroquinolones (7.1%) resistance, indicative of anthropogenic antibiotic use for growth promotion or prophylaxis (Mulchandani et al., 2023). Elevated cephalosporin resistance in captivity is particularly concerning, given the zoonotic potential of plasmid-borne *ESBLs* (Woerther et al., 2013).

The differential dominance of *rpoB2* (48.8% in grazing vs. 25.5% in captivity) further illustrates this dichotomy: grazing environments select for rifamycin-like resistance through natural antibiotic exposure (Wright and Poinar, 2012), while captivity favors efflux-based multidrug resistance. LEfSe analysis confirmed these patterns, with grazing enriched in environmentally acquired ARGs (*rpoB2*, *LlmA_23S_ribosomal*, *mupA*, *tet37*), while captivity favored efflux-associated determinants (*MexK*, *adeJ*, *abeM*). Notably, the enrichment of *adeJ*/*K* was associated with both antibiotic and biocide resistance (Andersson and Hughes, 2014), suggesting cross-selection driven by farm hygiene practices.

Host–microbiome associations revealed species-specific ARG signatures alongside functional convergence. For example, *rpoB2* (*Nocardia farcinica*) and *Bifidobacterium_adolescentis_rpoB* both confer rifamycin resistance via target alteration, reflecting evolutionary conservation (Spanogiannopoulos et al., 2016). *LlmA_23S_ribosomal* in *Paenibacillus* sp. *LC231* exemplifies ribosomal target modification, a strategy also observed in clinical pathogens such as *Staphylococcus* (Leclercq, 2002). Meanwhile, *ugd* in *Escherichia coli K-12* highlights peptide resistance via enzymatic target alteration (Baker et al., 2017). Efflux pumps such as *MexK (Pseudomonas aeruginosa)* and *adeJ*, *abeM* (*Acinetobacter baumannii*) underscore their role as broad-spectrum determinants in Gram-negative pathogens (Piddock, 2006). The unidentified host of *tet37* suggests environmental uncultured reservoirs (Forsberg et al., 2012), underscoring the importance of functional metagenomics to resolve novel resistance origins. We encountered challenges in assigning the *tet37* gene to any known organism, which suggests the possibility of an environmental reservoir for this gene. This limitation underscores the complexity of antibiotic resistance gene (ARG) dissemination in microbial communities and highlights gaps in our current understanding of ARG hosts. To address this, future research should use functional metagenomics approaches, which have the potential to uncover novel hosts and provide deeper insights into the ecological roles and dissemination pathways of ARGs (Sommer et al., 2009).

Our analysis suggests that resistome differences among captive sheep are indicative of anthropogenic selective pressure. However, a significant limitation of our study is the absence of antibiotic administration records, which restricts our ability to directly link specific antibiotics to observed ARG patterns. Consequently, our findings are drawn from ecological correlations rather than direct evidence of individual-level antibiotic exposure. This highlights the need for comprehensive records in future studies to better understand the relationship between antibiotic use and resistome composition.

## 5 Conclusion and implications

Our research demonstrated that feeding models exert strong ecological pressures to shape both microbial communities and resistomes in Awang sheep. Grazing favors resistance derived from environmental microbial reservoirs and natural antibiotics, while captivity selects for multidrug efflux and clinically relevant ARGs, likely due to antibiotic use and farm management practices. These results have direct implications for One Health frameworks: grazing systems may facilitate environmental ARG infiltration, while confinement amplifies clinically significant resistance, raising zoonotic risks. Future studies integrating cultivation-based approaches and host immune profiling will be essential to validate host–microbiome associations and evaluate potential transmission pathways.

### 5.1 Key findings

#### 5.1.1 Microbiome divergence

- Grazing animals exhibited higher abundances of fiber-degrading taxa (e.g., *Bacteroidetes* and *Ruminococcus*), reflecting adaptation to plant-rich diets.
- Confined herds showed enrichment of Proteobacteria (e.g., *Acinetobacter*) and multidrug efflux genes (e.g., *MexK*/*adeJ*), likely driven by concentrated feed, additives, or antimicrobial exposure.

#### 5.1.2 ARG landscape

- Grazing-associated ARGs: Dominated by *rpoB2* (rifamycin resistance) and peptide antibiotics, potentially linked to natural plant-derived compounds.
- Confinement-associated ARGs: Elevated clinical resistance (e.g., tetracyclines and macrolides) and plasmid-mediated efflux pumps, suggesting anthropogenic selection pressure.

#### 5.1.3 Ecological and practical implications

- The shift toward efflux-based resistance in confined systems may signal adaptive costs (e.g., energy expenditure) or horizontal gene transfer events.
- *Acinetobacter* and similar taxa could serve as biomarkers for monitoring AMR risks in intensive farming.

### 5.2 Future directions

- Mechanistic studies: Validate ARG–host associations (e.g., *Bifidobacterium*-derived *rpoB*) via culturomics.
- Functional assays: Couple metagenomics with metatranscriptomics to assess ARG expression dynamics.
- Intervention strategies: Evaluate dietary modifications (e.g., prebiotics) to mitigate high-risk ARGs in confined systems.

This study underscores the diet–microbiome–ARG axis as a critical lever for sustainable livestock production, balancing productivity with antibiotic stewardship.

## Data Availability

The datasets presented in this study can be found in online repositories. The names of the repository/repositories and accession number(s) can be found in the article/Supplementary material.

## Footnotes

1
https://www.python.org

2
https://www.r-project.org/

3
https://www.r-project.org/

4
https://www.r-project.org/

5
http://huttenhower.sph.harvard.edu/galaxy/root?tool_id=lefse_upload
